# The proportion of asymptomatic infections and spectrum of disease among pregnant women infected by Zika virus: systematic monitoring in French Guiana, 2016

**DOI:** 10.2807/1560-7917.ES.2017.22.44.17-00102

**Published:** 2017-11-02

**Authors:** Claude Flamand, Camille Fritzell, Séverine Matheus, Maryvonne Dueymes, Gabriel Carles, Anne Favre, Antoine Enfissi, Antoine Adde, Magalie Demar, Mirdad Kazanji, Simon Cauchemez, Dominique Rousset

**Affiliations:** 1 Epidemiology unit, Institut Pasteur in French Guiana, Cayenne, French Guiana; 2 National Reference Center for arboviruses, Institut Pasteur in French Guiana, Cayenne, French Guiana; 3 Laboratory, Centre Hospitalier Andrée Rosemon, Cayenne, French Guiana; 4 Gynaecology-Obstetrics Department, Centre Hospitalier de l’Ouest Guyanais, Saint-Laurent du Maroni, French Guiana; 5 Neonatology Department, Centre Hospitalier Andrée Rosemon, Cayenne, French Guiana; 6 Mathematical Modelling of Infectious Diseases Unit, Institut Pasteur, Paris, France; 7 Centre National de la Recherche Scientifique, URA3012, Paris, France; 8 Center of Bioinformatics, Biostatistics and Integrative Biology, Institut Pasteur, Paris, France; 9 These authors contributed equally to the study

**Keywords:** Zika virus, pregnant women, outbreak, epidemiology, French Guiana

## Abstract

Zika virus (ZIKV) infection has been associated with complications during pregnancy. Although the presence of symptoms might be a risk factor for complication, the proportion of ZIKV-infected pregnant women with symptoms remains unknown. Following the emergence of ZIKV in French Guiana, all pregnancies in the territory were monitored by RT-PCR and/or detection of ZIKV antibodies. Follow-up data collected during pregnancy monitoring interviews were analysed from 1 February to 1 June 2016. We enrolled 3,050 pregnant women aged 14–48 years and 573 (19%) had laboratory-confirmed ZIKV infection. Rash, arthralgia, myalgia and conjunctival hyperaemia were more frequently observed in ZIKV-positive women; 23% of them (95% confidence interval (CI): 20–27) had at least one symptom compatible with ZIKV infection. Women 30 years and older were significantly more likely to have symptoms than younger women (28% vs 20%). The proportion of symptomatic infections varied from 17% in the remote interior to 35% in the urbanised population near the coast (adjusted risk ratio: 1.6; 95% CI: 1.4–1.9.). These estimates put findings on cohorts of symptomatic ZIKV-positive pregnant women into the wider context of an epidemic with mainly asymptomatic infections. The proportion of symptomatic ZIKV infections appears to vary substantially between populations.

## Introduction

Zika virus (ZIKV) is a flavivirus that can be transmitted to humans by the bite of an infected *Aedes aegypti* mosquito, by sexual contact [1-3] or from mother to fetus [4]. Since the identification of ZIKV in Brazil in May 2015, the virus has spread rapidly throughout the Americas [5-10]. As at February 2017, 48 countries and territories of the region have reported active transmission of the virus [11]. Following this emergence, a number of studies showed that ZIKV infection in pregnant women was associated with congenital abnormalities such as microcephaly [4,7,12-18]. However, important discrepancies remain between the existing estimates of this risk, which was found to be substantially higher in pregnant women with symptomatic ZIKV infection [13] than in those with any ZIKV infection (i.e. symptomatic or asymptomatic infection) [17]. This suggests that the presence of symptoms might be a risk factor for complications [19,20]. In this context, risk assessment needs to rely on the proportion of asymptomatic infections among pregnant women infected by ZIKV. However, such description is currently lacking because published cohorts have so far focused on women with symptomatic ZIKV infections.

In French Guiana, a French overseas territory with 250,000 inhabitants located in the north-east of the southern American continent, the emergence of ZIKV has been considered to be of particular concern because the territory has the highest fertility rate in the Americas (3.5 children per woman), with an infant mortality rate (1.2%) that is three times higher than in metropolitan France (0.4%) [21]. On 22 January 2016, local health authorities launched an official epidemic alert following the rapid spread of ZIKV in the most inhabited part of the territory [22]. At the beginning of February, a territory-wide active monitoring system of all consenting pregnant women was implemented to report laboratory evidence of ZIKV infection during the outbreak. Here, we analyse data collected during the first 4 months of the outbreak to characterise the clinical manifestations of ZIKV infection in pregnant women, estimate the proportion of asymptomatic infections and study factors such as age and location that may affect the clinical presentation of ZIKV infection.

## Methods

### Study area

**Figure 1:**
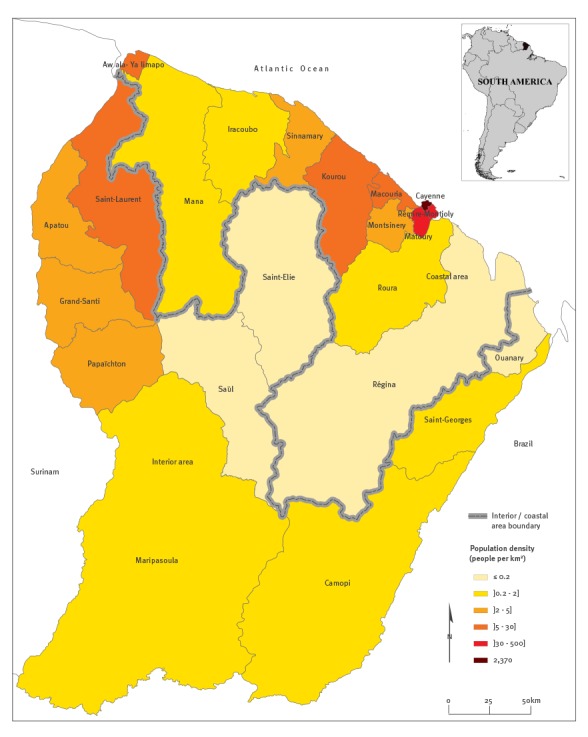
Map of French Guiana with the coastal/interior area and inhabitant densities

French Guiana, which is located in the Amazonian forest complex, is composed of two main inhabited geographical regions: a central urbanised and coastal strip area along the Atlantic Ocean where a large part of the population lives, and a more remote area along the Surinamese and Brazilian frontiers (Figure 1).

We will subsequently refer to these two areas as ‘the coastal area’ and ‘the interior’. The population of the coastal area is marked by a large variety of ethnic groups including Creoles (60% of the total population), people of European ancestry (14%) who essentially come from metropolitan France and various migrants from Brazil, Haïti, the Caribbean islands, China and southern Asia. The main groups living in the interior are the Maroons, descendants of escaped African slaves (15% of the total population of French Guiana) who live primarily in the western area, and the Amerindians (3%) who live preferentially in villages located in the southern interior part of the territory [23].

### Population cohort

The population cohort was recruited from the entire population of pregnant women seen in pregnancy consultations in French Guiana with recruitments starting at the beginning of February 2016 after the epidemic alert was emitted and the monitoring system was put in place. The analysis was performed with data available up to 1 June 2016.

### Ethical considerations

This analysis was based on data collected during the surveillance and response activities implemented during the ZIKV outbreak in French Guiana. All data used in this article were aggregated so that they could not be associated with any specific individual.

### Data collection 

Data were collected by clinicians or midwifes in charge of the pregnancy monitoring. Clinical, socio-demographic and geographical individual data were obtained at enrolment and at each pregnancy consultation by interviewing the pregnant women and from the medical histories.

For the entire pregnancy period that followed the start of the ZIKV epidemic, self-reported history of an acute febrile illness consistent with presumptive ZIKV infection was collected using a standardised questionnaire administrated during the consultation. Clinical characteristics including the occurrence of fever, macular or papular rash, petechiae, headache, myalgia, arthritis or arthralgia, conjunctival hyperaemia, nausea, haematoma, diarrhoea or epistaxis and date of onset were also collected by clinicians or midwifes responsible for the monitoring.

### Diagnosis of Zika virus infection

The monitoring includes a serological test at each trimester of pregnancy performed by the National Reference Center (NRC) for arboviruses in French Guiana with an in-house MAC-ELISA test (based on whole virus antigens obtained in cell culture and on hyperimmune ascitic fluid). The good performance of this ELISA test observed through systematic serological screening of a prospective ZIKV disease cohort [24] has been confirmed through the study of its diagnostic performance (unpublished data): the sensitivity, as evaluated on sera from 71 patients with ZIKV infection confirmed by real-time PCR and sampled between day 5 and day 20 after symptom onset, was 87% and increased to more than 98% for sera sampled after day 7 from symptoms onset. The specificity of the test varied greatly according to the panel used: it was very low in sera from people with confirmed acute dengue virus infection but increased to more than 80% for a panel of sera negative for all tested arboviruses (Zika, dengue, chikungunya) collected in French Guiana at the end of 2015 before the Zika epidemic and more than 2 years after the end of the last dengue epidemic. Finally, ZIKV neutralising antibodies have been found in all ZIKV IgM-positive samples (n = 33) tested by microneutralisation assay at the NRC for arboviruses from February to June 2016 (data not shown).

Serum and urine samples obtained during an acute illness or in the presence of structural abnormalities in the fetus or fetal death were assayed for ZIKV RNA by real-time RT-PCR using the Lanciotti method [25] or the RealStar ZIKV RT-PCR kit (altona Diagnostics GmbH, Hamburg, Germany). Analyses were performed by the National Reference Centre (NRC) for arboviruses at the Pasteur Institute in French Guiana for 2,906 samples (95%) and by the laboratory of the Cayenne Hospital Center for 144 (5%).

A ZIKV-positive woman was defined as a pregnant woman positive by real-time RT-PCR in at least one blood or urine sample and/or positive for IgM antibodies in serum irrespective of the IgG results. An asymptomatic ZIKV infection was defined as a positive ZIKV test in the absence of a documented ZIKV disease episode. A ZIKV-positive pregnant woman was considered to have a symptomatic ZIKV infection if she had a compatible clinical illness of ZIKV in the 7 days before confirmation for RT-PCR-confirmed cases or between the beginning of the outbreak and the date of laboratory diagnosis for IgM-positive cases. A compatible clinical illness was defined as at least one of the following symptoms: fever, a macular or papular rash, myalgia, arthralgia or conjunctival hyperaemia. The standard clinical case definition used for the surveillance system coordinated by French Public Health Agency was also applied to verify adequacy of the confirmation of ZIKV infection. A clinical case of ZIKV disease was defined as a person with a rash with or without fever and associated with at least two of the three following symptoms: conjunctival hyperaemia, arthralgia and myalgia without any other aetiology [9].

### Statistical analyses

Univariate analysis was performed using Fischer’s exact test for comparison between categorical variables. Poisson regression with robust error variance was used to identify factors associated with the presence of symptoms. The attributable fraction (AF) of symptoms among ZIKV-positive women was based on the risk ratio (RR) and calculated as AF_ZIKV_ = (RR−1)/RR [26]. Statistical analysis was performed using STATA12 software (Stata Corp., College Station, United States).

## Results

### Cohort description

Between 1 February and 1 June 2016, a total of 4,369 samples (3,951 sera and 418 urine samples) were collected from 3,050 pregnant women. Per pregnant woman, 1.4 samples were collected on average (range: 1–7), with a median time between two consecutive samples of 31 days (range: 1–129 days). Of the pregnant women included in the study, 1,489 (49%) were from the coastal area and 1,561 (51%) from the interior. The recruited women were 27.9 years-old on average (range: 14–48 years).

### Patients with Zika virus infection

**Figure 2:**
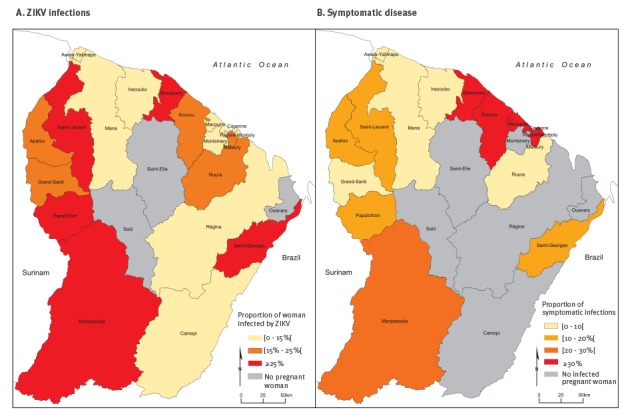
Proportion of pregnant women infected by ZIKV and proportion of symptomatic ZIKV infections, by area of residence, French Guiana, 1 February–1 June 2016 (n = 3,050)

A total of 573 women (18.8%) had laboratory evidence of ZIKV infection (Figure 2).

ZIKV: Zika virus.

Among them, 501 (87.4%) were IgM-positive and 96 (16.8%) were confirmed by RT-PCR. Twenty-four ZIKV-positive women (4.2%) were confirmed by both diagnostic assays. Among the 501 IgM-positive women, 456 (91.0%) were also IgG-positive. At the time of diagnosis, 24.6% (n=141) of the ZIKV-positive women were in the first trimester of pregnancy. The proportion of ZIKV infection peaked at 19.8% in March (Table 1). 

**Table 1:** Description and clinical characteristics of pregnant women, by ZIKV infection status, French Guiana, 1 February–1 June 2016 (n = 573)

Variable	Total n = 3,050		ZIKV-positive women n = 573		ZIKV-negative women n = 2,477		p value
	n	%	n	%	n	%	
**Month of inclusion **							
February	231	7.6	33/231	14.3	198/231	85.7	0.25
March	1,025	33.6	203/1,025	19.8	822/1,025	80.2	
April	1,069	35.0	205/1,069	19.2	864/1,069	80.8	
May	725	23.8	132/725	18.2	593/725	81.8	
**Age in years** ****Median [IQR]	3,050	100.0	28.0 [21.8 - 33.1]		27.5 [22.4 - 33.1]		NA
**Age groups**							
< 30 years old	1,864	61.1	337/1,864	18.1	1,527/1,864	81.9	0.22
≥ 30 years old	1,186	38.9	236/1,186	19.9	950/1,186	80.1	
**Area of residence **							
Interior	1,561	51.2	363/1,561	23.3	1,198/1,561	76.7	< 0.001
Coastal area	1,489	48.8	210/1,489	14.1	1,279/1,489	85.9	
**Presence of symptoms**	507	16.6	133/573	23.2	374/2,477	15.1	< 0.001
**Clinical characteristics**							
Fever	355	11.6	73/573	12.7	282/2,477	11.4	0.39
Rash	104	3.4	61/573	10.7	43/2,477	1.7	< 0.001
Arthralgia	184	6.0	56/573	9.8	128/2,477	5.2	< 0.001
Myalgia	185	6.1	56/573	9.8	129/2,477	5.2	< 0.001
Conjunctival hyperaemia	21	0.7	18/573	3.1	3/2,477	0.1	< 0.001
Headache	112	3.7	22/573	3.8	90/2,477	3.6	0.81
Nausea	15	0.5	4/573	0.7	11/2,477	0.4	0.50
Vomiting	10	0.3	2/573	0.4	8/2,477	0.3	1.00
Asthenia	14	0.5	4/573	0.7	10/2,477	0.4	0.32
Petechia	5	0.2	4/573	0.7	1/2,477	0.04	0.005
Diarrhoea	15	0.5	5/573	0.9	10/2,477	0.4	0.18

IQR: interquartile range; NA: not applicable.

The p value was calculated with Fisher’s exact test (two-sided).

A total of 513 women (89.5%) were confirmed for ZIKV at the inclusion in the monitoring system (i.e. with the first sample). The proportion of women infected by ZIKV was significantly lower in the coastal area (14.1%) than in the interior (23.3%; p < 0.001) (Figure 2A). It did not vary significantly with age.

### Proportion of symptomatic infections

The average delay between the reported date of symptom onset and the date of confirmation was 20.4 days (range: 1–116 days) for serology confirmation and 2.5 days (range: 0–9 days) for RT-PCR confirmation.

Overall, the proportion of ZIKV-positive women with at least one symptom compatible with a ZIKV infection was estimated at 23.2% (133/573; 95% confidence interval (CI): 19.7–26.7) (Table 2). Women aged 30 years or older were significantly more likely to have symptoms (28.0%; 95% CI: 22.3–34.1) than those younger than 30 years (19.9%; 95% CI: 15.7–24.5; p = 0.03)

**Table 2:** Factors associated with presence of symptoms among ZIKV-infected pregnant women, French Guiana February–May2016, French Guiana, 1 February–1 June 2016 (n = 573)

Variable	Total	Symptomatic cases		p value	Adjusted RR (95% CI)
		n	%		
Age group, years					
< 30	337	67	19.9	0.03	Ref
≥ 30	236	66	28.0		1.10 (0.94–1.29)
Area of residence					
Interior	363	60	16.5	0.001	Ref
Coastal area	210	73	34.8		1.64 (1.39–1.92)

CI: confidence interval; RR: risk ratio; ZIKV: Zika virus.

The p value is the global p value for the bivariate analysis calculated with Fisher’s exact test. Adjusted RR is the estimated multivariate risk ratio by Poisson regression with robust error variance.

The proportion of symptomatic infections was also significantly higher in the coastal area (34.8%; 95% CI: 28.3–41.2) than in the interior (16.5%; 95% CI: 12.7–20.4; p < 10^−3^). This difference remained significant after adjusting for age in a multivariate regression model (RR = 1.6; 95% CI: 1.4–1.9). The highest proportions were observed in the central part of the coastal area which contains the most populated municipalities of the territory (Figure 2B).

In the coastal area, the proportion of pregnant women reporting at least one symptom compatible with a ZIKV infection was 34.8% (73/210) among ZIKV-positive women and 18.5% (237/1,279) among ZIKV-negative women. Thus, the attributable fraction AF_ZIKV_ of symptoms due to ZIKV infection was 46.7% (95% CI: 33.7–57.1) in the coastal area. It was 30.8% (95% CI: 8.5–47.7) in the interior of the country.

### Clinical symptoms

Fever was the most frequently reported symptom in ZIKV-infected women (12.7%). However, presence of fever was also reported in 11.4% of ZIKV-negative women and was not significantly associated with ZIKV infection.

The symptoms listed in the Zika case definition that were significantly more prevalent in ZIKV-positive patients were rash (10.7% vs 1.7%, p < 0.001), arthralgia (9.8% vs 5.2%, p < 0.001), myalgia (9.8% vs 5.2%, p < 0.001) and conjunctival hyperaemia (3.1% vs 0.1%, p < 0.001) (Table 1).

Although petechiae were not included in the clinical case definition, we found that they were significantly associated with ZIKV infection (0.7% vs 0.1%, p = 0.005). Vomiting, nausea, asthenia, diarrhoea and other symptoms were rare (< 1%). We found that only 2.4% of ZIKV-positive women had symptoms that met the standard case definition for a clinical case of Zika disease vs 0.2% of ZIKV-negative women.

## Discussion

The introduction of ZIKV in French Guiana quickly triggered the implementation of systematic monitoring of all consenting pregnant women in the territory. In only four months, 3,050 pregnant women were included in the system, providing unique insights about ZIKV infection in pregnant women. Here, we analysed these data to characterise the spectrum of disease and the proportion of asymptomatic infections among pregnant women infected by ZIKV.

This systematic monitoring presents a number of interesting features. Firstly, it offers a more comprehensive and representative picture of the spectrum of disease following ZIKV infection in pregnant women than what would be obtained if recruitment had been based on a more specific criterion such as the presence of rash. It also makes it possible to estimate the proportion of asymptomatic infections that is particularly important in a context where symptoms might be associated with an increased risk of congenital complication [19,20]. Secondly, this approach made it possible to recruit in only 4 four months more than 3,000 pregnant women, of whom more than 500 had laboratory-confirmed ZIKV infection. This represents a substantial proportion of pregnancies in the territory (2,260 births were recorded during the study period) and is, to our knowledge, the largest cohort of ZIKV-infected pregnant women published so far.

We found that 19% of pregnant women enrolled in the study had at least one sample confirming ZIKV infection. We expect that this proportion underestimates the seroprevalence of ZIKV in the general population at the end of the study (1 June 2016) because samples were collected during the four previous months and women negative for IgM but positive for IgG were not considered as ZIKV-positive. Furthermore, pregnant women may have adopted protective behaviours and may therefore have been less affected by ZIKV than the general population.

The estimated proportion of 19% ZIKV-infected women is in contrast to the existing surveillance system that estimated around 7,000 consultations for ZIKV infection (2.8% of the population) by the end of the study [27]. This suggests that an important proportion of infections go unnoticed and highlights the benefits of testing subgroups of the population from routinely collected samples to monitor the risk of infection in the population and adjust the response accordingly. For example, although pregnant women from the interior area of the country had the highest levels of infection in our study, this area was never considered to be in the epidemic phase owing to the small number of clinical cases reported by the surveillance system.

The use of serological tools on repeated samples was particularly useful to define the infection status of pregnant women. Nineteen pregnant women were ZIKV-negative by real-time RT-PCR shortly after presenting symptoms but were later confirmed by serology. Even though some of these women might have been infected between the two assays, these findings suggest that the use of PCR as unique diagnosis tool may lead to false negative results. Although ZIKV antibody tests results are often difficult to interpret in endemic regions because of potential cross-reactivity with other flaviviruses [25,28], this is less of an issue in French Guiana in 2016 because there was no significant circulation of other flaviviruses in the 2 years before the ZIKV epidemic. During the study period, the NRC confirmed only four DENV cases among 1,460 individuals tested by RT-PCR and only 10 cases of confirmed dengue virus infection were reported by the health multi-source monitoring system between January and November 2016 [29]. Furthermore, among the ZIKV IgM-positive samples tested by microneutralisation test (n = 33), all contained ZIKV neutralising antibodies.

Most of the ZIKV epidemiological reports published so far have focused on symptomatic infections and clinical illness [7,9,13,30]. However, we found that 77% of pregnant women infected by ZIKV were asymptomatic. Since the presence of symptoms may affect the risk of congenital complication, it is essential that cohorts are set up to evaluate the risk of complication in both symptomatic and asymptomatic infections [19,20].

We also found that the proportion of symptomatic infections varied substantially over space, from 35% in the coastal area to only 17% in the interior area. A first hypothesis to explain these differences is that ethnicity affects the risk of developing symptoms following infection. While the population in the interior is mainly composed of Maroons, the population in the coastal area, particularly in the big municipalities, is more diverse, including Creoles, people of European ancestry and migrants from Asia and other parts of South America. The small proportions of symptomatic ZIKV infection in the interior may reflect, in part, difficulties in identifying rash on dark skin: among ZIKV-positive women, only 8% reported rash in the interior compared with 16% in the coastal area. However, we also observed significant differences between the two groups regarding fever, myalgia and arthralgia. Differences in historical exposure to other endemic flaviviruses such as dengue virus might explain theses discrepancies [31], although there is no seroprevalence data to validate or reject this hypothesis. In addition, we cannot rule out the possibility that individuals exposed to diseases and parasites in the most remote parts of the country are less likely to declare symptoms as a result of cultural, social and/or behavioural particularities. Nevertheless, results from the coastal area are consistent with those found during the 2007 ZIKV outbreak on Yap island in the Pacific Ocean, where 156 of 414 individuals (38%) who had IgM antibodies during household surveys reported a compatible illness during the outbreak period [32]. We found that rash, conjunctivis, myalgia and arthralgia were associated to ZIKV infection, which is consistent with previous studies [9,13,30,32,33]. The small proportion of ZIKV-positive women who had symptoms that met the standard clinical case definition advocates for an integrated epidemiologic surveillance system combining a non-specific but sensitive arboviral disease case definition with ZIKV positivity rates obtained from biological investigations.

The systematic monitoring of all consenting pregnant women in French Guiana came with a number of limitations. Firstly, to be efficient and readily acceptable in the whole territory, the questionnaire needed to be short and simple and could therefore not include the extensive list of variables that may be considered in more focused cohort studies. Secondly, recall bias may have led to under-reporting of symptoms, particularly when there is a considerable time delay between two consecutive consultations. However, the median time between consecutive consultations in our study was short (31 days; range: 1–129 days). Furthermore, since these delays did not vary between regions, they are unlikely to explain regional variations in the proportion of symptomatic infections. Finally, there may have been variations in the interview practices of individual clinicians and midwives.

The end of the ZIKV epidemic was declared on 9 September 2016 in some areas of French Guiana, with an estimated number of clinical cases close to 10,000 [34]. Transversal seroprevalence studies will be required to evaluate the full extent of the outbreak and to provide useful data to quantify the risks associated to ZIKV infection. Follow-up studies will focus on adverse consequences of the infection in fetuses and children of mothers with symptomatic or asymptomatic infection during pregnancy.

## Conclusion

This study described the spectrum of disease and the proportion of symptomatic infections in pregnant women infected by ZIKV. It also identified potential risk factors, such as age over 30 years and ethnicity, for symptomatic infection. In a context where the probability of birth defects could be affected by the presence of symptoms, such characterisation is important to support risk assessment of ZIKV infection in pregnancy.
